# Atypical thymic carcinoid tumor with ectopic ACTH syndrome in a 33-year-old male patient: A rare case report and literature review

**DOI:** 10.1097/MD.0000000000033847

**Published:** 2023-06-02

**Authors:** Le-Yao Li, Huan-Yu Zhao, Hai-Chao Tong, Ying-Chun Li, Hong-Tao Xu, Shuang Ma, Lian-He Yang, Wan-Lin Zhang, Tyler Wildes, Endi Wang

**Affiliations:** a Department of Pathology, the First Hospital and College of Basic Medical Sciences, China Medical University, Shenyang, China; b Department of Neurology, Sheng Jing Hospital of China Medical University, Shenyang, China; c Department of Pathology, Hebei Petro China Central Hospital, Langfang, China; d Department of Pathology, Duke University Medical Center, Durham, NC.

**Keywords:** ectopic ACTH syndrome, mediastinum, thymic neuroendocrine tumors

## Abstract

Atypical thymic carcinoid tumor is an exceedingly rare thymic neuroendocrine tumor derived from the cells of neuroendocrine system. Misdiagnosis or delayed diagnosis may result in disease progression to advanced stages and eventually leads to a poor prognosis. It is therefore necessary to make a correct diagnosis and provide an adequate treatment.

**Patient concerns:**

A 33-year-old Chinese male presented with numbness in bilateral lower extremities and general fatigue for a month. Chest computed tomography revealed a superior anterior mediastinal mass. Thymoma was initially considered, given the location of the mass and radiographic presentation.

**Diagnosis:**

Microscopic findings showed that the tumor cells are arranged in pseudoepitheliomatous growth or irregular nested growth pattern in a background of fibroconnective tissue, with focal infiltration into adipose tissue. The chrysanthemum-like structure or beam-like structure seen often in typical carcinoid tumor was not identified in this case. The tumor cells are spindled or oval, with focal active mitosis. The immunohistochemical staining showed strong positivity for CD56, CgA and Syn, positivity for CK, ACTH, and TTF-1, negativity for Vimentin, and ki67 labeled proliferation index was up to 10% in focal areas. According to the radiological and pathological findings, the diagnosis of atypical thymic carcinoid was made.

**Interventions:**

The patient underwent surgical resection of the mass.

**Outcome:**

No recurrence or metastasis was identified during the follow up.

**Lessons:**

Because of its low incidencen, onspecific clinical symptoms, tissue location, and radiological findings, atypical thymic carcinoid tumor may sometimes be misdiagnosed as thymoma. Attention should be paid to avoid misdiagnosis.

## 1. Introduction

Thymic neuroendocrine tumors are rare, accounting for 2% to 4% of interior mediastinal neoplasms,^[1]^ and 0.4% of all neuroendocrine tumors overall.^[2]^ These neoplasms exhibit local recurrence or distant metastasis in 20% to 30% of patients^[3]^ and usually occur in middle-aged males.^[2]^ Atypical carcinoid tumor is characterized by band, adenoid and solid nested cancer tissue with rosette-forming. Patients with atypical thymic carcinoid usually present with nonspecific symptoms. Therefore, atypical carcinoid tumor in thymus is likely to be diagnosed as thymoma from both histological and radiological impressions.^[4–7]^

Cushing’s syndrome is a set of clinical symptoms which occur as a result of hypercortisolemia. It usually results from overproduction of adrenocorticotropic hormone (ACTH) by a pituitary tumor, adrenal tumor, and adrenal hyperplasia. Ectopic syndrome related to an ACTH-secreting tumor represents 12% to 17% of Cushing’s syndrome cases.^[8]^ Ectopic ACTH production is mainly associated with small-cell carcinoma of the lung, but can also be found in various neuroendocrine tumors, such as bronchial, thymic, or pancreatic carcinoid, medullary carcinoma of the thyroid and pheochromocytoma. Atypical carcinoid tumor in thymus normally has no endocrine symptoms, and only some specific cases occur with carcinoid syndrome or with Cushing’s syndrome. Here, we reported a rare case of thymic atypical carcinoid tumor with ectopic ACTH syndrome occurred in 33-year-old Asian male.

## 2. Case presentation

A 33-year-old Asian male with 9-day history of poorly controlled hypertension treated with valsartan and amlodipine and a 7-day history of diabetes treated with acarbose was admitted to our hospital with the complaint of numbness in both lower extremities and fatigue. Recently, the patient suffered from dizziness without headache, blurred vision, nausea and vomiting.

**Figure 1.**
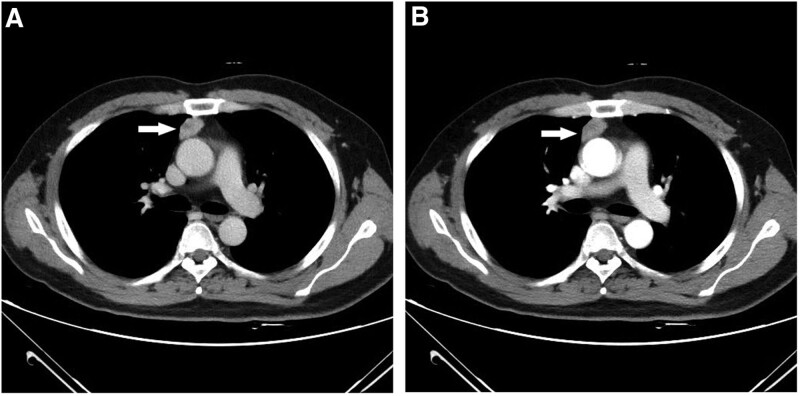
Pre-operative CT scan of the mediastinum. (A–B) the mediastinal CT showing a soft tissue lesion in anterior superior mediastinum with homogenous enhancement. CT = computed tomography.

A computed tomography (CT) scan included the plain scan of the lung and the plain scan + enhanced CT of the mediastinum revealed a nodular dense shadow in the anterior mediastinum, indicating a lesion. The following mediastinal CT result showed an irregular dense shadow (2.1 × 2.9 cm) protruding to one side of lung field with a sharp edge and soft tissue lesion in the anterior superior mediastinum with homogenous enhancement (Fig. 1A and B). This result indicated that the space-occupying lesion might be thymoma with an atypical carcinoid tumor in mediastinum accompanied with ACTH ectopic syndrome.

**Figure 2.**
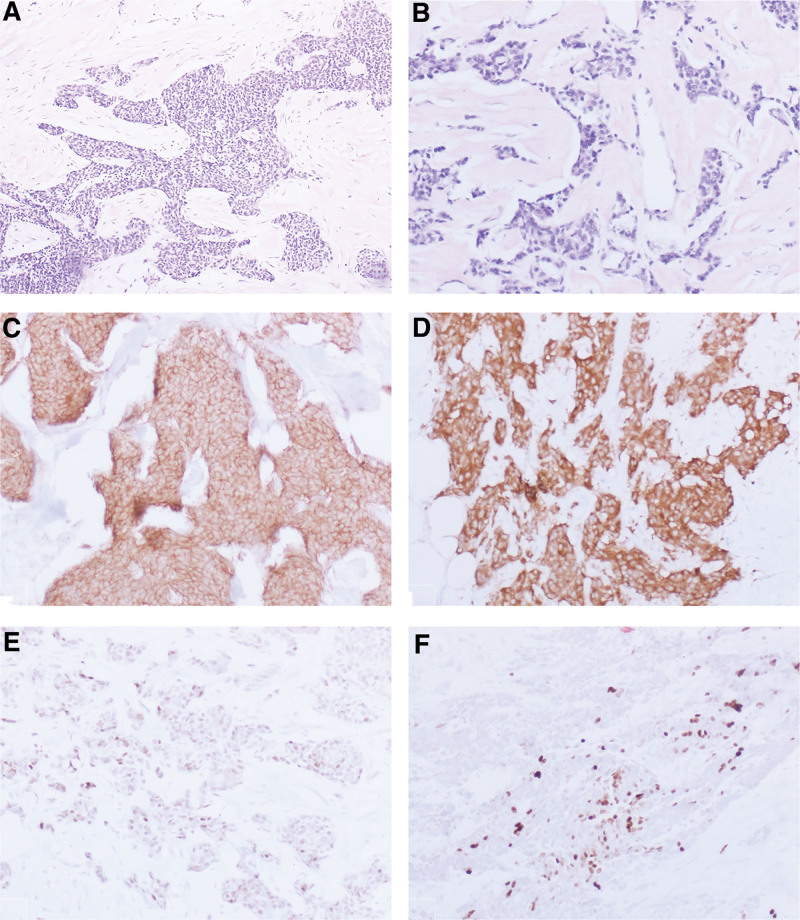
(A) The tumor cells are arranged in irregular sheets and solid nests (Hematoxylin and eosin stain ×100); (B) the neoplasm cells are medium-sized and uniform, with round or oval nuclei, fine granular chromatin and no obvious nucleoli (Hematoxylin and eosin stain ×200); (C) the tumor cells revealed positive expression of CD56(immunohistochemistry ×200); (D) the tumor cells showed positive expression of synaptophysin (immunohistochemistry ×200); (E) the tumor cells revealed partially positive expression of TTF-1 (immunohistochemistry ×200); (F) The Ki67 index is about 20% in the hot spot (immunohistochemistry ×200).

The lesion was removed through thoracoscopic resection and considered as malignant tumor during surgery. The lesion was diagnosed as atypical carcinoid tumor during routing histopathological analysis. Microscopic appearance showed tumor cells arranged in solid or irregular nested arrangements, with local infiltration into adipose tissue (Fig. 2A). The arrangement of tumor cells lacks the chrysanthemum-like structure or beam-like structure in typical carcinoids The tumor cells were spindle-shaped or oval, with active local mitoses (Fig. 2B. 5/10 high-power field). The lesion was diagnosed as atypical carcinoid tumor. The immunohistochemical staining showed CD56, CgA, and Syn strong positive (Fig. 2C and D); CK, ACTH, TTF-1 positive; Vimentin negative; ki67 index of local hot spot about 10% positive (Fig. 2E and F), which supported the diagnosis.

## 3. Discussion

Thymic neuroendocrine tumors mostly refer to thymic carcinoid tumor, which originate from thymic neuroendocrine cells. About one-third of patients with atypical thymic carcinoid tumors are asymptomatic,^[9]^ so its diagnosis mainly depends on pathological examination. A few patients have presented with chest pain and superior vena cava symptoms owing to the specific position of tumor in anterior mediastinal.^[10]^ Thymic NETs can be categorized into 2 types: well-differentiated NETs comprising typical carcinoids and AC, and poorly differentiated NETs comprising large cell and small cell neuroendocrine carcinoma. Immunohistochemical tests also aid in identifying these tumors types, differentiating more benign carcinoids from more aggressive carcinomas. Approximately 50% of patients with thymic carcinoid have endocrine disorders, most of which is Cushing’s syndrome due to ectopic ACTH production or multiple endocrine neoplasia syndrome, specifically Type-1 multiple endocrine neoplasia.^[11]^ Patients with ACTH ectopic syndrome often express moon-shaped faces, central obesity, livid striae, hypokalemia with amyosthenia, hypertension, and hyperlipidemia. The patient in our case demonstrated most of the above symptoms. After the lesion in the mediastinum was removed surgically, the ACTH level precipitously decreased. Therefore, we anticipate that the ACTH ectopic syndrome was caused by the functional thymic atypical carcinoid tumor in this case.

Imaging examinations are not specific for the diagnosis of atypical carcinoid tumors of the thymus. According to Shimamoto et al,^[12]^ thymic carcinoids tend to be large masses with irregular contours, no capsule, heterogeneous intensity on T2 weighted images, heterogeneous enhancement and local invasion on CT or magnetic resonance imaging. A necrotic or cystic component was often seen in AC. Pathological examination is the main diagnostic examination of the atypical thymus carcinoid. In this case, we found an index TTF-1 was positive, while normally the index is negative in mediastinal atypical carcinoid tumor. TTF-1 is mainly expressed in glandular epithelium of the thyroid and epithelial cells of the lung, which can be used to identify pulmonary adenocarcinoma and squamous carcinoma.

The differential diagnosis of the patients with interior mediastinal lesions includes all kinds of primary mediastinal tumors, including epithelial thymoma, parathyroid tumors, lymphoma and metastatic tumors.

Treatment options of thymic carcinoids are surgical excision, chemotherapy, and radiotherapy, of which surgery is the most effective treatment for thymic NETs.^[13–16]^ The prognosis is better in patients with surgery than those with conservative treatment, with a 5-year survival rate of 58%.^[17]^ Patients with thymic neuroendocrine tumors have a poor prognosis because of the aggressive nature of atypical carcinoid tumors that have high rates of recurrence and metastasis. The overall 5-year survival rate is 30% to 70%,^[3,18–20]^ and the overall 10-year survival rate is 30%.^[21]^ Therefore, early diagnosis is essential to improve the prognosis of atypical thymic carcinoid.

In summary, atypical carcinoid tumor in the mediastinum, especially with ACTH ectopic syndrome and TTF-1 positivity is very rare. Clinical symptoms are not specific. Here we reported a case of atypical carcinoid tumor in the mediastinum with ACTH ectopic syndrome that was suspected to be thymoma through CT. After surgery, it was confirmed as atypical carcinoid tumor. We reviewed the morbidity, symptoms, radiographic features and differential diagnosis of atypical carcinoid tumor in the mediastinum which may be helpful in clinical practice. Because of its morbidity, attention should be paid to avoid misdiagnosis.

## Author contributions

**Conceptualization:** Ying-Chun Li, Shuang Ma, Lian-He Yang, Endi Wang.

Data curation: Leyao Li.

Funding acquisition: Shuang Ma, Lian-He Yang.

Investigation: Leyao Li, Hai-Chao Tong, Ying-Chun Li, Wan-Lin Zhang.

Methodology: Leyao Li, Hai-Chao Tong, Endi Wang.

Project administration: Lian-He Yang.

Resources: Wan-Lin Zhang.

Software: Wan-Lin Zhang.

Supervision: Huan-Yu Zhao, Hong-Tao Xu, Tyler Wildes, Endi Wang.

Validation: Hong-Tao Xu, Tyler Wildes.

Visualization: Leyao Li.

Writing – original draft: Leyao Li.

Writing – review & editing: Huan-Yu Zhao, Hong-Tao Xu, Shuang Ma, Lian-He Yang.
